# P-1513. Immunogenicity and Safety of Bivalent Respiratory Syncytial Virus (RSVpreF) Vaccine in Non-Pregnant HIV Infected Older Adults

**DOI:** 10.1093/ofid/ofaf695.1697

**Published:** 2026-01-11

**Authors:** Gonzalo Perez Marc, Tarek Mikati, Vishva Bangad, Daniel P Eiras, John Woodside, Michael Patton, Kumar Ilangovan, David Radley, Maria Maddalena Lino, Elena Kalinina, Kena A Swanson, Annaliesa S Anderson, Alejandra C Gurtman, Iona Munjal

**Affiliations:** i-trials, Buenos Aires, Ciudad Autonoma de Buenos Aires, Argentina; 3. Pfizer, Inc., Vaccine Research & Development, Pearl River, NY; Pfizer, Inc., Cincinnati, Ohio; Pfizer, Inc., Cincinnati, Ohio; Pfizer, London, England, United Kingdom; Pfizer, Vaccine Research and Development, Hurley, England, United Kingdom; Vaccine Research and Development, Pfizer, USA, Raleigh, North Carolina; Pfizer, London, England, United Kingdom; Pfizer, London, England, United Kingdom; Pfizer, London, England, United Kingdom; Pfizer, London, England, United Kingdom; Pfizer, London, England, United Kingdom; Pfizer, London, England, United Kingdom; Pfizer, Inc., Cincinnati, Ohio

## Abstract

**Background:**

People living with HIV (PLWHIV) are at increased risk of severe respiratory syncytial virus (RSV) disease due to their weakened immune status and higher prevalence of cardiopulmonary diseases. United States (US) HIV guidelines recommend RSV vaccination to those at risk of severe RSV infection; however, there is limited data on immune responses after RSV vaccine receipt among this population. PLWHIV were included in the RSVpreF clinical trials, and the objective of this analysis is to assess the safety and immunogenicity among the subset of PLWHIV participants from the pivotal Phase 3 Study in which efficacy was established in older adults (≥ 60 years) immunized against RSV disease (“RENOIR” trial NCT 050035212).

**Figure 1:**
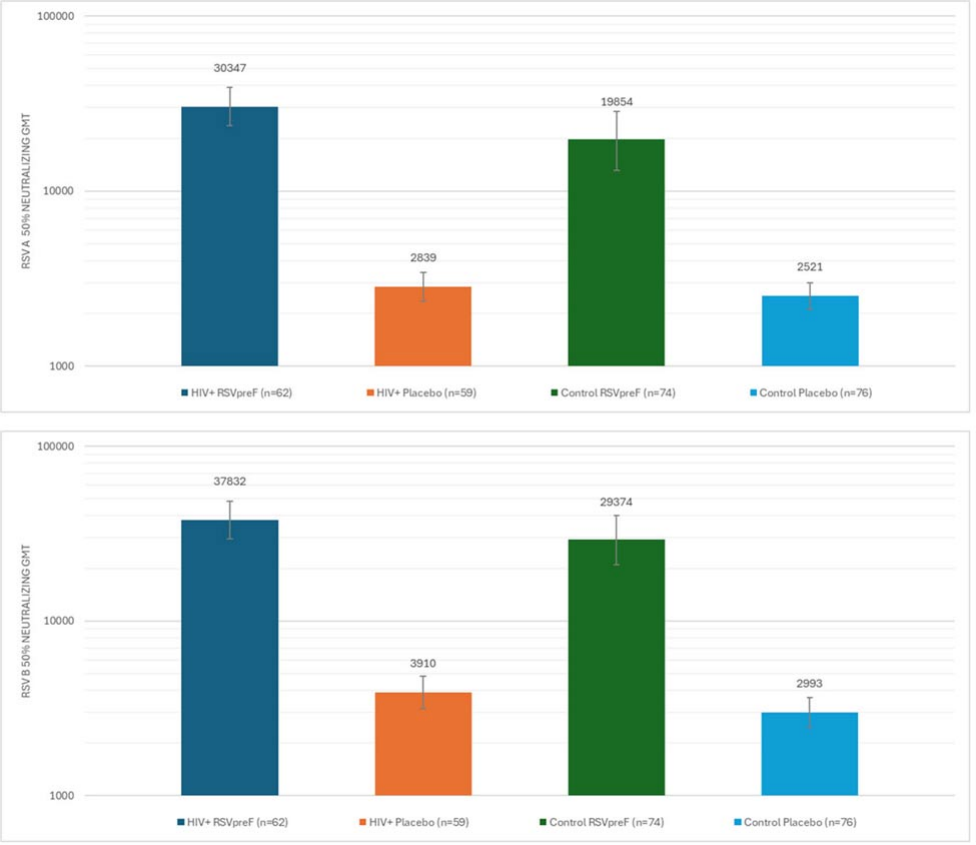
RSV Neutralizing GMT in Participants with HIV and Controls by Vaccine Group

**Table 1:**
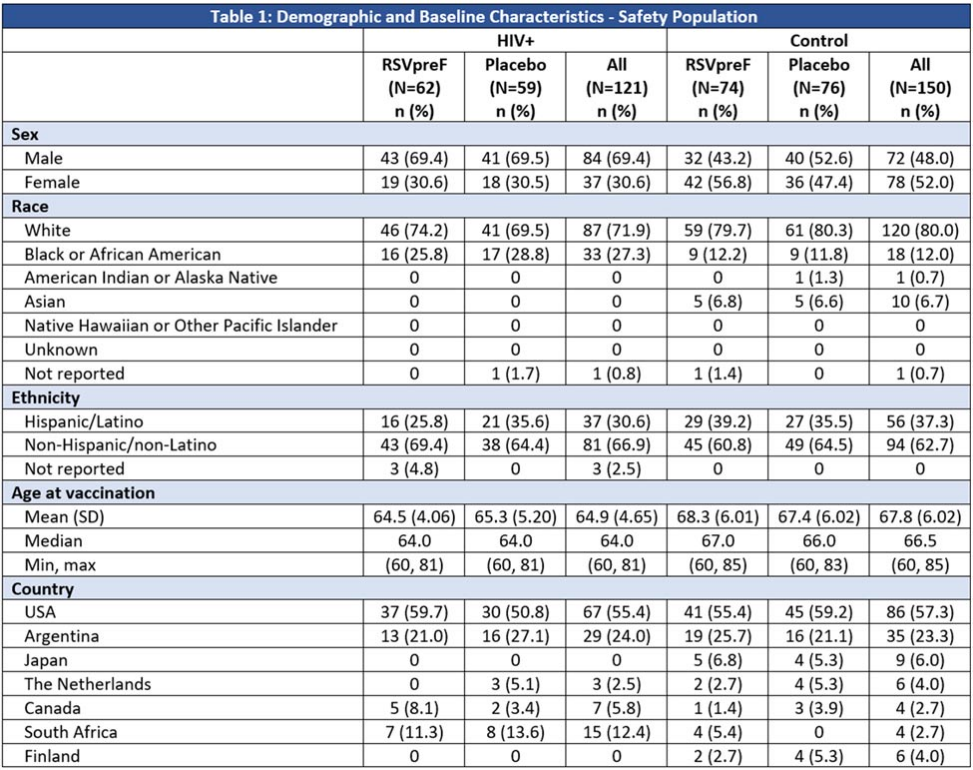
Demographic and Baseline Characteristics - Safety Population

**Methods:**

To assess the safety and immunogenicity of bivalent RSVpreF among RENOIR participants with a medical history of well-controlled HIV, participants were identified and their blood samples collected one month post study intervention were assessed for RSV neutralizing geometric mean titers (GMT). A control group without a medical history of HIV was randomly selected for comparison. Safety was assessed in all subjects meeting the criteria for selection.

**Table 2:**
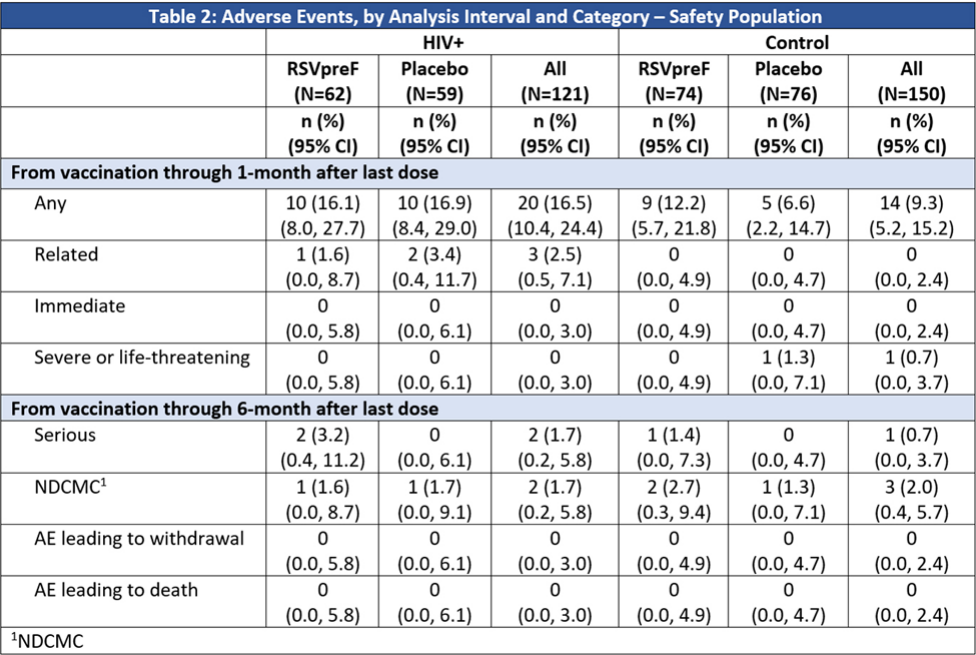
Adverse Events, by Analysis Interval and Category – Safety Population

**Results:**

Among 121 PLWHIV included in safety and immunogenicity analyses, proportions of the subgroup population were balanced between receipt of RSVpreF or placebo (Table 1). The participants were mainly from the US, followed by Argentina and South Africa. Compared to the control group, there were a higher proportion of males, Black race and younger age participants in the PLWHIV group (Tables 1). Safety events were reported similarly among RSVpreF and placebo recipients in the PLWHIV subgroup, though more frequently than the control; none were severe or life threatening (Table 2). Post-vaccination RSV neutralizing GMTs in the PLWHIV were 9-10-fold higher in RSVpreF recipients compared to placebo recipients; similar neutralizing titer trends were observed in the control (non-HIV) group (Figure 1).

**Conclusion:**

Overall, vaccination with bivalent RSVpreF elicited a robust immune response in adults ≥60 years with a medical history of well controlled HIV. No safety signals were identified in this subgroup.

**Disclosures:**

Gonzalo Perez Marc, MD, Boehringer-Ingelheim: Grant/Research Support|Enanta: Advisor/Consultant|GSK: Grant/Research Support|Merck: Grant/Research Support|Moderna: Grant/Research Support|Pfizer, Inc.: Advisor/Consultant|Pfizer, Inc.: Grant/Research Support|Pfizer, Inc.: Honoraria|Sanofi: Grant/Research Support|Sequiris: Honoraria Tarek Mikati, MD,MPH, Pfizer, Inc.: Salary|Pfizer, Inc.: Stocks/Bonds (Public Company) Vishva Bangad, MS, Pfizer, Inc.: Salary|Pfizer, Inc.: Stocks/Bonds (Public Company) Daniel P. Eiras, MD, MPH, Pfizer, Inc.: Salary|Pfizer, Inc.: Stocks/Bonds (Public Company) John Woodside, PhD, Pfizer, Inc.: Salary|Pfizer, Inc.: Stocks/Bonds (Public Company) Michael Patton, B.Sc., Pfizer, Inc.: Salary|Pfizer, Inc.: Stocks/Bonds (Public Company) Kumar Ilangovan, MD, MSPH, MMCi, Pfizer, Inc.: Salary|Pfizer, Inc.: Stocks/Bonds (Public Company) David Radley, MS, Pfizer, Inc.: Salary|Pfizer, Inc.: Stocks/Bonds (Public Company) Maria Maddalena Lino, PhD, Pfizer, Inc.: Salary|Pfizer, Inc.: Stocks/Bonds (Public Company) Elena Kalinina, PhD, Pfizer, Inc.: Stocks/Bonds (Public Company) Kena A. Swanson, Ph.D., Pfizer, Inc.: Salary|Pfizer, Inc.: Stocks/Bonds (Public Company) Annaliesa S. Anderson, PhD, Pfizer Inc: Employee Alejandra C. Gurtman, M.D., Pfizer, Inc.: Salary|Pfizer, Inc.: Stocks/Bonds (Public Company) Iona Munjal, MD, Pfizer, Inc.: Salary|Pfizer, Inc.: Stocks/Bonds (Public Company)

